# The Sulfate-Reducing Bacteria Hypothesis in Parkinson’s Disease: From *Desulfovibrio*-Derived Hydrogen Sulfide to Alpha-Synuclein Aggregation

**DOI:** 10.3390/biomedicines14102256

**Published:** 2026-10-06

**Authors:** Michał Koterba, Aleksandra Skiba, Anna Kler, Karolina Błaszczyk, Monika Woźny, Oliwia Stefaniak, Joanna Nowicka

**Affiliations:** 1 Student Scientific Society of Microbiologists, Wroclaw Medical University, 50-368 Wroclaw, Poland; aleksandra.skiba@student.umw.edu.pl (A.S.); monika.wozny@student.umw.edu.pl (M.W.); oliwia.stefaniak@student.umw.edu.pl (O.S.); 2 Department of Microbiology, Faculty of Medicine, Wroclaw Medical University, 50-368 Wroclaw, Poland

**Keywords:** Parkinson’s disease, *Desulfovibrio*, hydrogen sulfide, alpha-synuclein, gut–brain axis, vagus nerve, gut microbiome

## Abstract

The conceptualization of Parkinson’s disease (PD) has shifted from a strictly central neurodegenerative disorder to a systemic synucleinopathy that may, in some patients, originate in the enteric nervous system. This review synthesizes recent evidence highlighting the potential role of sulfate-reducing *Desulfovibrio* (DSV) bacteria as candidate pathobionts in the proposed “gut-first” pathogenic cascade. By colonizing anaerobic niches within the colon and metabolizing dietary sulfur compounds, DSV generate hydrogen sulfide (H_2_S), a gaseous metabolite that may compromise intestinal barrier integrity and impair mitochondrial function through the inhibition of cytochrome c oxidase. These processes have been associated with ATP depletion, cytochrome c release, and iron-dependent oxidative stress, creating conditions that may favor α-synuclein misfolding and aggregation within enteroendocrine cells. We further examine evidence supporting the retrograde propagation of α-synuclein pathology from the gut to the brain via the vagus nerve—a hypothesis strengthened by experimental findings and epidemiological observations, including studies reporting reduced PD risk following truncal vagotomy. Although the increased prevalence of DSV in PD patients suggests potential applications in prodromal diagnostics and microbiome-based risk stratification, a definitive causal relationship has yet to be established. We therefore discuss key methodological limitations, including biases associated with 16S rRNA sequencing and the current lack of robust in vivo metabolomic evidence. Finally, we consider whether targeted modulation of the intestinal environment, including DSV-specific bacteriophages and [FeFe]-hydrogenase inhibitors, could emerge as a future strategy for influencing early disease processes.

## 1. Introduction: The Shift from Brain-First to Gut-First Paradigm

### 1.1. The Evolution of Understanding PD as a Systemic Disease

Parkinson’s disease (PD) is a slowly progressive neurodegenerative disorder and the second most common neurodegenerative disease worldwide. The incidence increases with age, currently affecting an estimated 8.5 million people globally, and that number is steadily rising [1,2]. The disease is characterized by the loss of cells in the dopaminergic neurons in the substantia nigra and striatum, as well as the formation of α-synuclein (α-syn) aggregates that form intraneuronal Lewy bodies in both the central and peripheral nervous systems [3].

The classic motor symptoms of PD include bradykinesia, limb rigidity, and resting tremor. In addition to the above, there are also non-motor symptoms, including autonomic dysfunction, sleep disturbances, pain, and neuropsychiatric symptoms. These features usually appear long before motor symptoms develop (prodromal phase, premotor phase). Some of the most common, earliest symptoms of PD include constipation and gastrointestinal disorders, such as dysphagia, gastroparesis, nausea, and heartburn [1,3].

The contemporary perception of PD as a systemic disease is reflected in evolving diagnostic approaches. Current clinical diagnostic criteria, which are based on the assessment of clinical symptoms, apply only in the advanced stages of the disease, when 60–80% of the dopaminergic neurons in the substantia nigra have already been lost—a process that occurs 5–15 years after the onset of underlying neuronal degeneration [4]. The growing knowledge of the pathophysiological basis of PD, combined with advances in the study of disease biomarkers in vivo, is driving a shift in the diagnostic paradigm from a clinical to a biological one. This shift in approach could potentially enable the diagnosis of the disease in its early stages, when classic clinical symptoms have not yet appeared [3].

There are two primary forms of PD: familial and sporadic. The familial form is caused by genetic abnormalities, including mutations in the α-syn gene (point mutations A30P, A53T, E46K, H50Q, and G51D, or duplication or triplication of the locus). Conversely, the cause of sporadic PD remains unknown. Research points to both genetic factors and environmental exposure to toxic substances, including pesticides (rotenone and paraquat) and the neurotoxin MPTP [5]. The main factors contributing to the development of the disease are believed to be the misfolding and aggregation of α-syn, leading to the formation of Lewy bodies—intracellular aggregates of abnormal, insoluble α-syn—in both the central and peripheral nervous systems, as well as neuroinflammation and oxidative stress [6].

### 1.2. The Braak Hypothesis Revisited

The hypothesis proposed by Braak et al. in 2003 highlights the significant role of the gastrointestinal tract in the pathogenesis of sporadic PD [7]. This hypothesis posits the existence of an unknown pathogen (such as virus or bacterium) that induces the abnormal folding and aggregation of α-syn. The pathological process initially occurs in the dorsal motor nucleus of the glossopharyngeal or vagus nerve, or in the anterior olfactory nucleus. Subsequently, less sensitive gray matter nuclei and cortical areas are gradually affected. This is followed by involvement of the cerebral cortex, beginning with the anteromedial temporal mesocortex. From there, the pathology spreads to the neocortex, starting with higher-order sensory association and prefrontal areas. This is followed by first-order sensory-association/pre-motor areas and primary sensory–motor fields. Propagation occurs via an intercellular transfer mechanism similar to prion transmission [5,7].

The presence of gastrointestinal disorders, such as dysphagia, nausea, constipation, and gastrointestinal motility disorders, in patients with PD long before the onset of motor symptoms may support Braak’s hypothesis. It is estimated that approximately 80% of patients with PD suffer from constipation [8]. The introduction of the gut–brain hypothesis led Horsager et al. to propose an alternative hypothesis positing the existence of two subtypes of PD: a top-down “brain-first” subtype, in which α-syn pathology initially arises in the brain and then spreads to the peripheral autonomic nervous system, and a bottom-up “body-first” subtype, in which the pathology originates in the gut or peripheral autonomic nervous system and then spreads to the brain [6,9,10].

### 1.3. The Search for the “Smoking Gun”

The presence of α-syn aggregates in both the substantia nigra and the enteric nervous system (ENS) has drawn attention to a potential link between the gut microbiota and PD [6]. The collection of microorganisms both symbiotic and pathogenic that inhabit the bodies of humans and other animals, known as the microbiota, consists of up to 100 trillion bacteria (as well as certain fungi, archaea, and viruses), with a total mass of 1 to 2 kg. The total genome of the gut microbiota contains approximately 3 million genes, which is 150 times larger than the human genome [8]. Through interactions within the intestinal microenvironment between the epithelium, the mucosal immune system, and the ENS, the microbiota regulates the physiology of the gastrointestinal tract. In turn, the ENS communicates with the central nervous system via the gut–brain axis (GBA), a bidirectional network connecting the gut to the brain [6,11].

Dysbiosis, or a disturbance in the qualitative and quantitative composition of the gut microbiota, is associated with diet, medications, the intestinal mucosa, and the immune system. Factors that exacerbate the shift leading to dysbiosis include oxidative stress, bacteriophages, and bacteriocins [12]. Comparative studies of the gut microbiota in patients with PD reveal significant alterations compared with healthy individuals. Notably, an increase in the abundance of the genera *Lactobacillus* and *Bifidobacterium* has been observed. Some studies indicate a decrease in the levels of the families *Faecalibacterium*, *Coprococcus*, and *Blautia*. Stool sample analyses have revealed an increased abundance of *Akkermansia* and *Bifidobacterium* and a decrease in the abundance of *Lachnospiraceae* and *Faecalibacterium* [13,14]. Numerous studies also indicate that the microbiota evolves across the different stages of PD. A correlation with disease progression has been observed, among others, with a decline in *Bifidobacterium* and *Bacteroides fragilis*, *Prevotella* and *Prevotellaceae*, and *Puniceicoccaceae*. For *Escherichia* and *Shigella*, a negative correlation with disease duration was demonstrated, whereas *Klebsiella* showed a positive correlation. However, these studies do not account for differences in microbiota composition related to the subjects’ varying dietary habits and other factors that may influence microbiota architecture [10,13].

Another group of bacteria whose overgrowth is observed in PD includes hydrogen sulfide (H_2_S)-producing species, such as *Helicobacter pylori* and *Clostridium difficile*, as well as mucus-associated species from the genera *Desulfovibrionaceae* and *Enterobacteriaceae*. It has been demonstrated that patients with PD exhibit significantly higher concentrations of H_2_S in their cerebrospinal fluid compared to the control group [15]. It has been suggested that excessive H_2_S production may have a detrimental effect on human cells by inducing mitochondrial cytochrome c release, increasing iron levels, and promoting the generation of reactive oxygen species (ROS), which in turn may lead to the formation of α-syn oligomers and fibrils in cells containing the α-syn protein. Furthermore, H_2_S produced by bacteria may disrupt urate metabolism and affect erythrocytes and lymphocytes in the blood [16]. Recent studies indicate a strong correlation between the severity of PD and the concentration of *Desulfovibrio* (DSV) bacteria. Furthermore, all stool samples from PD patients tested positive for the DSV-specific [FeFe] hydrogenase gene. Constipation, one of the prodromal symptoms of PD, also correlates strongly with the concentration of DSV [17].

### 1.4. Objectives

This review aims to synthesize recent evidence highlighting the critical role of *Desulfovibrio* species in the pathogenesis of Parkinson’s disease. Specifically, it explores the mechanistic pathways through which excessive bacterial hydrogen sulfide production and subsequent mitochondrial dysfunction initiate the misfolding of α-synuclein within the ENS. Furthermore, this paper critically evaluates the retrograde transport of α-synuclein aggregates via the vagus nerve and discusses the clinical implications of these findings, including potential diagnostic biomarkers and targeted therapeutic interventions.

## 2. *Desulfovibrio* as a Unique Pathobiont in the PD Gut

### 2.1. Niche Ecology

The colonic environment is a highly competitive anaerobic ecosystem where various microbial groups compete for limited metabolic substrates. Among these, sulfate-reducing bacteria (SRB), particularly DSV, occupy a unique ecological niche defined by their specialized energy metabolism [18]. Unlike most gut commensals that rely on fermentation, DSV utilize sulfate as a terminal electron acceptor in a process known as dissimilatory sulfate reduction [19]. In this pathway DSV oxidize organic acids or molecular hydrogen (H_2_) to reduce sulfate to H_2_S [20].

In the anaerobic environment of the large intestine H_2_ is a primary byproduct of carbohydrate fermentation. Competition for H_2_ is a critical determinant of microbial community structure. DSV exhibit a significantly higher affinity for H_2_ than methanogens or acetogens, especially when sulfate concentrations are non-limiting [21]. This thermodynamic advantage allows DSV to outcompete other hydrogenotrophic microbes, establishing them as a dominant force in the terminal stages of organic matter degradation [18]. However, the byproduct of this metabolic activity, H_2_S, is a potent cytotoxin. While low levels of H_2_S may serve as signaling molecules, excessive production characteristic of DSV expansion can impair colonocyte respiration by inhibiting cytochrome c oxidase and compromise the integrity of the intestinal mucosal barrier [22,23].

### 2.2. Dietary Drivers

The abundance and metabolic potency of DSV are strongly dependent on host dietary habits, as the “Western diet” provides an optimal substrate profile for DSV proliferation [24]. Such eating habits, characterized by high intakes of animal products and processed foods, increase the supply of both inorganic and organic sulfur-containing compounds to the colon [25]. Sulfates of inorganic origin are frequently used as additives (e.g., carrageenan and sulfites) in processed foods feeding directly into the SRB metabolic route [26].

In addition, the intake of red meat is a major source of sulfur-containing amino acids and taurine. Taurine, which is conjugated to bile acids, escape ileal reabsorption and reach the colon, where DSV have a specific enzymatic system to deconjugate and use it as a both carbon and sulfur source [27]. The Western diet also contains a high amount of heme, which has been reported to specifically stimulate DSV growth, while promoting oxidative stress in the gut lumen, favoring these pathobionts [28].

### 2.3. Iron Interaction

One essential element of DSV pathogenicity is the complex manipulation of host iron metabolism. DSV iron acquisition is very efficient including the production of siderophores which are able to outcompete host proteins for access to ferric iron [20]. This sequestration facilitates enzymatic demands of the bacteria itself, many of which are iron–sulfur (Fe-S) cluster proteins intricately involved in the sulfate reduction pathway and also perturbs local iron availability in the gut lumen [20].

The Fenton reaction is closely associated with DSV-induced oxidative stress. H_2_S produced by DSV is also capable of converting ferric iron (Fe^3+^) to the more reactive ferrous iron (Fe^2+^), which then interacts with hydrogen peroxide to form highly reactive hydroxyl radicals [29]. This results in lipid peroxidation and damage to the intestinal epithelium [30]. Also, emerging evidence shows that long-term colonization with DSV may modulate systemic metal bioavailability and contribute to the iron dyshomeostasis observed in the substantia nigra seen of patients with PD [31]. By changing the gut redox state and consuming essential metals, DSV represent a continual stimulus for systemic oxidative challenges.

### 2.4. Prevalence in PD

Recent clinical data have consistently reported an abundance of DSV in fecal microbiota of patients with PD. University of Helsinki-led research has shown that DSV occur in virtually all PD patients and, in many cases, at significantly higher relative abundances compared with age-matched healthy controls [32]. Importantly, the amount of these bacteria correlates with disease progression: patients with higher DSV levels more often show more severe symptoms and faster disease progression [32].

The DSV presence is not only a marker of dysbiosis, but is also considered an agent that contributes to the accumulation of α-syn. Using *Caenorhabditis elegans*, experimental models have shown that DSV strains derived from PD patients induce misfolding and aggregation of α-syn, a pathological hallmark of PD [16]. Since DSV-mediated H_2_S generation can contribute to disruption of the blood–brain barrier (BBB) and trigger systemic inflammation, their designation as a “pathobiont” offers a mechanistic explanation for the link between gut health and neurodegeneration [31,33]. This association supports the implication of DSV as a feasible diagnostic biomarker and therapeutic target. While early gastrointestinal dysfunctions, such as constipation, are known to strongly affect the composition of the gut microbiome [34], it remains unclear whether the increased abundance of DSV is a cause or a consequence of these early symptoms. Regardless of the exact initial trigger, the developing intestinal inflammation exerts a strong selective pressure on the microbial community [35]. Therefore, the proliferation of *Desulfovibrio* in Parkinson’s disease patients is not random, but rather driven primarily by intensified homogeneous selection and weakened stochastic drift within this inflamed intestinal microenvironment. These evolutionary processes cause the microbiome profiles of patients to become more deterministic and closely correlated with clinical severity stages according to the Hoehn and Yahr scale [36]. The connection between DSV proliferation and PD pathology warrants further investigation in the context of molecular level colonization of the host’s ENS by these bacteria.

## 3. The Molecular Mechanism: Hydrogen Sulfide (H_2_S) and Mitochondrial Toxicity

### 3.1. The Janus Face of H_2_S

H_2_S is currently recognized as a gasotransmitter, a class of endogenously produced gaseous signaling molecules that includes nitric oxide (NO) and carbon monoxide (CO). These molecules are enzymatically generated, freely permeable across biological membranes, and exert tightly regulated, dose-dependent biological effects [37]. Under physiological conditions, H_2_S contributes to the regulation of inflammation, maintenance of vascular tone, and modulation of immune responses [38].

At low to moderate concentrations, H_2_S exerts predominantly cytoprotective effects. It supports mitochondrial function, enhances antioxidant defenses, and contributes to cellular homeostasis [38,39]. One of its key roles involves maintaining mitochondrial integrity through stimulation of autophagic processes that remove damaged mitochondria and cellular debris. Efficient autophagic flux reduces oxidative stress and prevents the accumulation of dysfunctional organelles. Conversely, impaired H_2_S production may compromise autophagy, increasing susceptibility to oxidative damage and contributing to disease progression. This relationship is particularly important in neurons, which rely heavily on mitochondrial function and are therefore especially vulnerable to disruptions in mitochondrial quality control [39,40].

The antioxidant effects of H_2_S are mediated primarily through indirect mechanisms. Although H_2_S can react with certain reactive species, its physiological concentrations are insufficient for substantial direct radical scavenging. Instead, H_2_S regulates redox homeostasis by activating endogenous defense systems. It promotes activation of redox-sensitive transcription factors such as Nrf2, leading to increased expression of antioxidant enzymes including superoxide dismutase (SOD), glutathione peroxidase (GPx), and heme oxygenase-1 (HO-1) [39]. Additionally, H_2_S supports maintenance of intracellular thiol pools, particularly glutathione (GSH), which is essential for detoxification of ROS [39,40]. Furthermore, H_2_S may reduce ROS generation by modulating pro-oxidant enzymes such as NADPH oxidases. Taken together, these mechanisms indicate that H_2_S serves primarily as a regulator of redox balance rather than a direct radical scavenger [39,40].

Despite these protective roles, the biological effects of H_2_S are highly concentration-dependent. While nanomolar to low micromolar concentrations are associated with cytoprotection, excessive accumulation shifts toward cytotoxicity [39]. Elevated H_2_S levels have been linked to mitochondrial dysfunction, inflammation, and tissue damage [38]. This shift depends on tissue-specific factors, including metabolic demand, oxygen availability, and the efficiency of enzymatic systems responsible for H_2_S metabolism, such as sulfide quinone oxidoreductase (SQR) [39]. This is particularly relevant in the brain, where even subtle disturbances in mitochondrial function and redox balance may lead to significant pathological consequences [38,39]. However, the precise concentration thresholds at which H_2_S shifts from a cytoprotective to a cytotoxic molecule remain poorly defined and may vary between tissues [39].

Collectively, these observations support a model in which microbiota-derived H_2_S acts as a central mediator linking mitochondrial dysfunction, iron dysregulation, and oxidative stress, thereby creating conditions that favour α-syn misfolding and aggregation [38,39,40].

### 3.2. Disruption of Mucosal Integrity

The concentration-dependent effects of H_2_S are also evident in the gastrointestinal tract, where it plays a critical role in maintaining mucosal homeostasis. However, excessive H_2_S production, particularly under conditions of gut dysbiosis, can compromise intestinal barrier integrity.

Overproduction of H_2_S by SRB, especially species of the genus *Desulfovibrio* (DSV), has been associated with increased intestinal permeability, commonly referred to as “leaky gut” [38]. Elevated luminal H_2_S disrupts the mucus layer by reducing disulfide bonds within the mucin-2 network, leading to structural destabilization of the epithelial barrier [41]. As a result, pro-inflammatory molecules such as lipopolysaccharides (LPS) can translocate into the bloodstream, triggering systemic inflammation. This increased permeability represents a key mechanistic link between gut dysbiosis and both systemic and neuroinflammatory responses, including those implicated in neurodegeneration [38].

When colonocytes exhibit a reduced capacity to detoxify H_2_S via the sulfide oxidation unit (SOU), intracellular H_2_S levels rise further. This accumulation impairs mitochondrial ATP production and is accompanied by increased expression of inflammatory mediators such as interleukin-6 (IL-6) and inducible nitric oxide synthase (iNOS), thereby amplifying local inflammation [41]. Experimental models have shown that high concentrations of microbiota-derived H_2_S contribute to mucin degradation and further weaken the epithelial barrier [38].

The biological effects of H_2_S also depend on its source. While endogenously produced H_2_S in epithelial cells may exert protective effects, excessive bacterial production promotes inflammation and barrier dysfunction [42]. However, complete inhibition of endogenous H_2_S synthesis is detrimental, as basal levels are necessary to maintain mucosal integrity [41]. Together, these findings highlight that both insufficient and excessive H_2_S levels may disrupt intestinal homeostasis, emphasizing the need for tightly regulated H_2_S metabolism [38,41,42].

### 3.3. Inhibition of Cytochrome C Oxidase

At elevated concentrations, H_2_S directly targets mitochondrial function by inhibiting cytochrome c oxidase (Complex IV) of the electron transport chain. H_2_S binds to the heme–copper center of the enzyme, blocking electron transfer to molecular oxygen and thereby inhibiting the final step of oxidative phosphorylation [39,43,44,45].

This inhibition leads to a rapid decline in ATP production, resulting in cellular energy deficiency. Experimental studies have demonstrated that exposure to high concentrations of H_2_S can significantly reduce Complex IV activity, accompanied by structural and functional mitochondrial alterations [46]. This ATP depletion directly impairs ATP-dependent cellular processes, including protein folding, degradation, and the maintenance of proteostasis [39].

This toxicity is primarily attributed to the binding of H_2_S to cytochrome c oxidase, leading to respiratory inhibition [44]. However, H_2_S can also act as an electron donor via SQR, highlighting its dual and context-dependent interaction with mitochondrial respiration [45].

Inhibition of oxidative phosphorylation not only limits ATP production but also promotes electron leakage from the electron transport chain, resulting in increased ROS generation. Thus, H_2_S exposure produces a dual-hit effect marked by concurrent energy failure and oxidative stress, both of which are central features of neurodegeneration [39,40].

### 3.4. Oxidative Stress and the Fenton Reaction

Mitochondrial dysfunction and increased ROS production create conditions that favor pathological protein aggregation, including α-syn in PD [39,40]. H_2_S contributes to this process through interconnected mechanisms involving mitochondrial damage, iron dysregulation, and oxidative stress.

Elevated H_2_S levels induce the release of cytochrome c (Cyt c) from mitochondria into the cytosol [15]. In neurons, this release does not necessarily trigger apoptosis but instead may lead to acquisition of peroxidase activity by Cyt c, particularly in the presence of anionic lipids. In this state, Cyt c can utilize hydrogen peroxide to promote oxidative modifications of α-syn, facilitating its aggregation. This suggests that mitochondrial dysfunction may promote α-syn aggregation independently of classical apoptotic pathways [15,47].

H_2_S has been shown to disrupt cellular iron homeostasis, increasing the labile iron pool and promoting iron-dependent oxidative stress. Available evidence suggests that H_2_S may enhance the availability of redox-active Fe^2+^, thereby facilitating ROS-generating reactions, although the precise molecular mechanisms remain poorly defined. This effect is likely mediated through indirect redox interactions rather than a single defined pathway of iron release [15,48].

Elevated Fe^2+^ levels promote the Fenton reaction, generating highly reactive hydroxyl radicals that drive lipid peroxidation and cellular damage [48]. Consequently, H_2_S amplifies oxidative stress not only through mitochondrial dysfunction but also by functionally increasing the pool of catalytic iron required for ROS generation. This convergence of mitochondrial ROS production and iron dysregulation generates a highly pro-oxidative intracellular environment [15,48]. This iron-dependent oxidative damage is consistent with ferroptosis, a form of regulated cell death increasingly implicated in PD pathology [48]. Importantly, whether such levels of labile iron and H_2_S are consistently achieved in vivo remains to be fully established [15,48].

Excess iron further promotes α-syn aggregation and impairs autophagic clearance by inhibiting autophagosome–lysosome fusion [15]. In parallel, iron-mediated oxidative damage disrupts mitochondrial membranes and electron transport chain function, leading to further ATP depletion [48]. Additional factors, such as magnetite nanoparticles derived from DSV species, may further enhance ROS production [15]. The coexistence of α-syn, ROS, and Cyt c creates a highly reactive intracellular environment that promotes protein aggregation. These aggregates may propagate in a prion-like manner along the gut–brain axis, potentially reaching the central nervous system via the vagus nerve [15].

Altogether, these processes establish a self-amplifying cycle in which H_2_S-induced mitochondrial dysfunction, iron dysregulation, and oxidative stress mutually reinforce each other, ultimately promoting α-syn aggregation [15,48].

### 3.5. Role of S-Sulfhydration

In contrast to the predominantly toxic mechanisms described above, H_2_S also exerts regulatory effects through post-translational protein modification known as persulfidation (S-sulfhydration), in which cysteine residues are converted to persulfide groups (–SSH) [37,45]. This modification can alter protein activity and enhance cytoprotective pathways, playing a crucial role in mitochondrial quality control.

A key target of this modification is Parkin, an E3 ubiquitin ligase involved in the clearance of damaged mitochondria via mitophagy. Persulfidation enhances Parkin activity, promoting the removal of dysfunctional mitochondria and maintaining cellular homeostasis [45]. In PD, decreased persulfidation of Parkin has been observed, leading to impaired mitophagy and the accumulation of damaged mitochondria [37]. This impairment further limits the cell’s ability to control oxidative stress and maintain mitochondrial integrity, thereby exacerbating neurodegenerative processes [37,45].

Under physiological conditions, H_2_S-mediated persulfidation reduces oxidative stress, prevents mitochondrial dysfunction, and inhibits apoptosis. However, disruption of this regulatory mechanism contributes to mitochondrial impairment and disease progression [45].

These combined mitochondrial and redox disturbances create conditions that favor the initiation of α-syn misfolding and aggregation in PD.

## 4. Triggering Alpha-Synuclein Aggregation: Evidence from Recent Studies

### 4.1. From Oxidative Stress to Misfolding

α-syn is an intrinsically unstable protein prone to misfolding and aggregation [49,50]. While proper folding is required for its physiological function, structural destabilization promotes the formation of misfolded species that progressively accumulate into oligomeric and insoluble aggregates [49]. Recent evidence indicates that the intestinal microenvironment may initiate these pathological processes. DSV species are frequently detected in fecal samples of patients with PD, and their abundance correlates with disease severity. The presence of DSV-specific [FeFe]-hydrogenase genes further suggests the involvement of diverse and potentially uncharacterized strains in disease pathogenesis [32].

Mechanistically, intestinal colonization by DSV leads to increased H_2_S production, which may exceed the detoxification capacity of the mucosal sulfide oxidation pathway. In parallel, DSV produce LPS and other bioactive factors in close proximity to enteroendocrine cells, contributing to neuroinflammatory responses [50]. Together, these factors create conditions that favor α-syn misfolding and aggregation within intestinal cells [32,50]. Misfolded α-syn may subsequently spread in a prion-like manner to the central nervous system via the vagus nerve and systemic circulation, contributing to dopaminergic neuronal dysfunction. Recent metagenomic studies (MAGs) show that strains, like *Desulfovibrio* sp. MAG58, have assimilatory and almost complete dissimilatory sulfate reduction pathways. This makes them more likely to produce H_2_S. These pathways are found in amounts, in advanced stages of Parkinson’s disease. The more these pathways are present the worse the motor symptoms are as measured by the MDS-UPDRS scale [36]. Bacterially derived factors may further enhance aggregation once they reach the brain [32]. At the cellular level, DSV promote α-syn aggregation both directly and indirectly through soluble mediators released from affected intestinal cells. One proposed mechanism involves the activation of Toll-like receptor 2 (TLR-2), which enhances α-syn expression and accumulation via downstream signaling pathways. In addition, DSV may impair intracellular clearance mechanisms, including lysosomal pathways, leading to protein accumulation. H_2_S-mediated mitochondrial dysfunction, associated with cytochrome c release and elevated ROS, further promotes oligomerization. These stress conditions may also promote the release of α-syn into the extracellular space, enabling its uptake by neighboring cells and amplifying aggregation. This process is reinforced by proinflammatory mediators such as TNF-α, linking aggregation with neuroinflammation [50].

Importantly, microbiome-associated effects depend on variables such as exposure duration and host susceptibility. The presence of phosphorylated pS129 α-syn underscores the pathological relevance of these processes. However, in wild-type models, they likely represent early disease stages rather than fully developed pathology [51].

### 4.2. The Helsinki Breakthrough

To evaluate the ability of DSV strains to induce α-syn aggregation, a transgenic *C. elegans* model expressing human α-syn fused to a fluorescent reporter was used. Nematodes were exposed to DSV strains isolated from PD patients and healthy individuals, alongside *Escherichia coli* controls representing curli-producing (positive) and non-producing (negative) variants. Confocal microscopy enabled quantitative and qualitative assessment of aggregation. Exposure to PD-derived DSV strains resulted in a significantly higher number of α-syn aggregates compared with both healthy-derived strains and controls, demonstrating a clear cause and effect relationship. Although strains from healthy individuals also induced aggregation, the effect was markedly weaker, indicating strain-dependent pathogenicity.

Volumetric analysis further showed that PD-derived strains induced not only more aggregates but also larger ones, reflecting increased proteotoxic burden. This effect varied between species, with *Desulfovibrio desulfuricans* and *Desulfovibrio fairfieldensis* promoting larger aggregates than *Desulfovibrio piger*, highlighting heterogeneity in virulence traits. These differences translated into distinct biological outcomes, as worms exposed to DSV—particularly PD-associated strains—exhibited increased mortality.

Mechanistically, these effects are consistent with H_2_S-mediated oxidative stress and mitochondrial dysfunction, which promote α-syn aggregation. Variability in H_2_S production may arise from differences in hydrogenase systems and enzymatic profiles. In addition, DSV adhesion to and invasion of intestinal epithelial cells may impair barrier integrity, promoting inflammation and microbial translocation, which further amplifies aggregation. Structurally diverse LPS molecules may also contribute to strain-specific toxicity. Notably, α-syn aggregation was modulated in a strain-dependent manner. *D. desulfuricans* DSM 6949 and the clinical isolate *Desulfovibrio* spp. MUU 26 induced the highest aggregation burden, suggesting enhanced pathogenic potential. This observation aligns with the broader concept that clinical isolates often exhibit increased virulence.

Collectively, these findings demonstrate that DSV strains differentially influence α-syn aggregation and toxicity, supporting a mechanistic connection between gut microbiota composition and neurodegeneration. They also highlight the need for validation in mammalian models and for comparative genomic studies to identify determinants of DSV-associated pathogenicity in PD [16,52].

### 4.3. Comparison with Other Pathobiont Models

α-syn aggregation arises from converging microbiota-driven pathways involving both inflammatory and aggregation-promoting bacterial factors. DSV species and curli-producing bacteria such as *Escherichia coli* represent complementary yet mechanistically distinct models of this process. A shared upstream feature is the induction of a proinflammatory intestinal environment. Bacterial components, including LPS and amyloidogenic factors, trigger immune activation that may progress to chronic low-grade inflammation, increased intestinal permeability, and systemic dissemination of inflammatory mediators, potentially compromising the BBB [32]. These conditions facilitate α-syn misfolding.

Despite this common background, the mechanisms driving aggregation differ substantially. In curli-producing *E. coli*, aggregation is primarily mediated by structural templating. Curli fibers, composed of CsgA and CsgB subunits with a cross-β-sheet architecture, act as bacterial amyloids that directly promote α-syn fibrillization via cross-seeding [10,52,53]. Experimental data show that CsgA accelerates fibril formation and increases deposition in vivo, supporting direct interaction between bacterial and host amyloids [10]. Immune activation further amplifies this process as both amyloid types are recognized by similar receptors [53]. The specificity of this mechanism is demonstrated by the absence of aggregation in curli-deficient *E. coli* strains [54].

In contrast, DSV-associated aggregation is driven predominantly by chemically mediated mechanisms. DSV-derived metabolites, particularly H_2_S, disrupt cellular homeostasis by inducing mitochondrial dysfunction, increasing ROS, and altering iron metabolism, thereby promoting α-syn misfolding [15]. Unlike curli, these factors do not directly seed aggregation but instead create intracellular conditions that favor protein instability and impaired proteostasis. Although DSV may produce amyloid-like proteins, this mechanism remains less well characterized [50]. Their primary impact appears to stem from diffusible metabolites such as H_2_S, which can cross biological barriers and exert systemic effects, including within the central nervous system. Together with circulating inflammatory mediators, these factors may facilitate the propagation of toxic α-syn species via neural and hematogenous routes [15].

Importantly, inflammation alone is insufficient to induce aggregation. Other bacteria, such as *Proteus mirabilis* or *Klebsiella pneumoniae*, can promote intestinal inflammation but do not consistently trigger α-syn aggregation, underscoring the importance of specific bacterial properties [54]. Taken together, DSV represent a distinct pathobiont model in which aggregation is driven primarily by metabolic and redox-mediated mechanisms, in contrast to the structurally driven cross-seeding observed in curli-producing bacteria. These pathways converge on α-syn misfolding but differ in their initiating factors, highlighting multiple, complementary microbiota-related mechanisms in PD pathogenesis. To summarize these distinct pathways, Table 1 provides a comprehensive mechanistic comparison of how *Desulfovibrio* spp., curli-producing *E. coli*, and general pro-inflammatory bacteria differentially contribute to α-syn aggregation.

The emergence of α-syn aggregation within the intestinal environment raises the question of how these pathological species access the central nervous system, with increasing evidence pointing to retrograde transport along the vagus nerve.

## 5. Retrograde Transport: The Vagal Highway to the Midbrain

### 5.1. ENS as “Ground Zero”

The origin of oligomeric α-syn aggregates has long remained an open issue, despite significantly elevated levels being observed in the blood plasma of patients with PD. Braak’s hypothesis proposes that PD pathogenesis may originate in the gastrointestinal tract, with the vagus nerve (VN) serving as a “highway” for the propagation of pathological α-syn from the gut to the brain [55]. Supporting this hypothesis, experimental evidence shows that different forms of α-syn injected into the intestinal wall are transported to the brainstem via the vagal nerve [15]. The VN and the dorsal motor nucleus of the vagus (DMV) form a likely route for pathology to spread from the ENS to the Central Nervous System (CNS), where neurons transmit these potentially toxic oligomers to one another via their axons [5]. Recent research identifies DSV bacteria as a key initiator at “ground zero”, as these sulfate-reducing bacteria significantly increase α-syn expression and aggregation within intestinal enteroendocrine cells (EECs) [50]. These bacteria may provoke local inflammation and oxidative stress through the production of H_2_S, which releases cytochrome c from mitochondria and leads to α-syn accumulation [16]. Furthermore, DSV-induced impairment of the intestinal barrier, or “leaky gut,” allows α-syn aggregates and bacterial products to enter the systemic circulation or the vagal pathway, where they may eventually seed further aggregation in the brain and trigger a vicious circle of neuroinflammation [5,50].

### 5.2. Prion-like Cell-to-Cell Propagation

The progression of neurodegenerative disorders such as Alzheimer’s disease (AD) and PD is driven by a prion-like molecular process known as corruptive templating or template-directed aggregation [56]. This mechanism begins when normally produced, soluble cellular proteins undergo a conformational change into insoluble, *β*-sheet-rich species that self-assemble into proteopathic “seeds” [56,57]. Once established, these aberrant proteins propagate by binding to similar molecules and acting as structural templates that induce native proteins to adopt the same pathogenic conformation [58]. This crystallization-like process allows the assemblies to grow, fragment, and proliferate, effectively amplifying the pathological agent throughout the nervous system [57,58]. For these proteinaceous lesions to spread non-randomly throughout the brain, the seeds must translocate between cells and brain regions, a process primarily mediated by the neural connectome, in which active axonal transport along defined pathways serves as a major driver of dissemination [58]. Experimental evidence indicates that fibrillar forms of proteins such as amyloid-*β*, tau, and α-syn are internalized by neurons and conveyed via both anterograde and retrograde transport [56,58]. In “peripheral-first” scenarios, such as the “gut-to-brain” hypothesis, seeds are taken up at peripheral axon terminals and transported retrogradely to the central nervous system, whereas “brain-first” scenarios involve anterograde spread from vulnerable brain regions to secondary sites [56,59]. Beyond traditional axonal trafficking, intercellular transfer is facilitated by exosomal release in microvesicles, direct transfer through F-actin-based tunneling nanotubes, and uptake via active or passive endocytosis, often mediated by specific receptors such as LAG3 [56,57,58]. While neuronal pathways are the primary route of propagation, non-neuronal cells such as microglia and astrocytes also participate in the uptake and processing of seeds [56,58]. However, the efficiency of this propagation is constrained by intrinsic biological barriers. For instance, the extracellular matrix can act as a physical barrier to uptake, and the success of templated conversion is ultimately governed by the structural compatibility, or “seed-soil” interaction, between the pathogenic seed and the host’s endogenous protein substrate [56,58,60].

### 5.3. Vagal Denervation Studies

The potential for α-syn pathology to spread to the brain via the NV remains a central focus of PD research, providing preliminary and indirect support for Braak’s dual-hit hypothesis, which proposes that PD pathology can originate in the gut and spread retrogradely to the brain via prion-like mechanisms [61,62,63]. At the cellular level, recent evidence demonstrates that pathological human α-syn can transfer from gut mucosal cells, specifically EECs, to interconnected nerves, supporting a non-neuronal source for of abnormal α-syn [64]. Because EECs are positioned at the critical interface between the gut lumen and enteric nerve fibers, they are directly exposed to the microbiome, microbial metabolites, and ingested toxicants such as pesticides, making them a highly plausible site for the initial formation and subsequent propagation of misfolded proteins to adjacent neurons and the vagal ganglia [64]. While animal studies suggest that vagotomy disrupts the propagation of α-syn from the gut to the central nervous system, findings from human epidemiological studies have been conflicting [65]. In a large, population-based study, researchers found no association between overall vagotomy and PD risk but observed a significantly lower risk of PD specifically among patients who underwent truncal vagotomy, a procedure that denervates multiple gastrointestinal organs where Lewy pathology typically appears [61]. In contrast, selective or highly selective vagotomies, which denervate only parts of the stomach, showed no protective effect, presumably because synucleinopathy could still find an intact pathway to the brainstem through other gastrointestinal sites [61]. This protective association of truncal vagotomy was stronger 5 to 10 years after surgery but attenuated after more than 20 years, suggesting that truncal vagotomy may merely delay rather than eliminate PD risk [61]. This attenuation is consistent with the possibility that PD pathology can originate at multiple sites within the peripheral nervous system, such as the submandibular gland, or the olfactory bulb [61].

Furthermore, alternative explanations for these findings cannot be ruled out, as the vagus nerve also plays a critical role in the cholinergic anti-inflammatory pathway capable of suppressing local and systemic inflammation. It remains unknown how truncal vagotomy alters these inflammatory responses and affects cellular mechanisms responsible for handling α-syn aggregates [62]. Ultimately, the extent to which this gut-to-brain spread is a key causal step in PD remains to be established, and it is likely to be only one of several “highways” or mechanisms involved in initiating the neurodegenerative process [63]. Other gut–brain connections are also emerging, including the potential role of gut-associated lymphoid tissue (GALT), supported by evidence that tonsillectomy may protect against PD, and sensory pathways linking the upper gut to striatal dopamine release [63]. Consequently, further research, preferably using prospective study designs, is needed to clarify the precise molecular mechanisms of cell-to-cell transfer and the true protective role of vagotomy in the development of PD [64,65].

## 6. Methodological Challenges: Why We Might Be Missing the Full Picture

### 6.1. The 16S rRNA Bias

Current research on the gut microbiome in patients with PD relies heavily on 16S rRNA gene sequencing. However, this method has significant limitations in taxonomic resolution. Standard universal primers often cannot distinguish between closely related DSV strains. Since the pathogenic effects associated with PD may vary by strain, grouping these bacteria into a single taxonomic unit obscures important biological details [32]. As noted by Menozzi et al. [66], these simplified approaches add to the inconsistencies in describing the GBA and fail to identify the specific microbial populations that contribute to disease progression [67].

### 6.2. Culturomics vs. Sequencing

High-throughput sequencing provides a comprehensive profile of microbial community composition; however, it does not determine the functional activity of the identified organisms [35,67]. DSV species, as sulfate-reducing bacteria, possess highly specific environmental requirements, requiring strictly anoxic conditions for growth [32]. Consequently, a methodological approach known as “culturomics” is needed to evaluate whether the detected microorganisms exhibit pathogenic potential [16,66]. This method, however, presents significant technical challenges.

A major limitation arises from the fact that microorganisms identified via sequencing cannot consistently be cultured under laboratory conditions. This constraint impedes the definitive establishment of a causal relationship between specific DSV species and α-syn aggregation [16]. Without viable cultures, evidence for the mechanistic role of DSV in modulating the ENS remains largely indirect and associative [35].

### 6.3. Metabolomics Gap

The role of SRB in PD is even more difficult to determine because of the challenge in quantifying H_2_S in vivo. H_2_S is a reactive, volatile signaling molecule that is swiftly oxidized or bound by host tissues at the time of production. Fecal H_2_S levels do not accurately reflect the site-specific concentrations within the intestinal lumen, where DSV come into contact with the mucosal barrier. Menozzi et al. [66] highlight that the current inability to accurately measure such transient metabolic signals remains a major hindrance in conceptualizing the GBA and its therapeutic applications. These technical barriers require novel, real-time sensing methods to successfully link microbial presence to functional disease pathology.

### 6.4. Future Perspectives: The Role of Multi-Omics Approaches

To fix the limits of sequencing methods modern research is using newer metagenomic approaches and shotgun metagenomics. These methods let scientists spot microbes at the strain level and see what they do inside communities [68]. Also new high-resolution metabolomics can measure different metabolic signs at once. This gives clues about how microbes influence the gut–brain connection [66]. When researchers combine these multi-omics tools they can link the presence of microbes to disease functions. They also uncover metabolic messages that push neurodegeneration forward [66]. As a result these high-resolution techniques help find biomarkers and design precise treatments for Parkinson’s disease [68].

## 7. Clinical Implications: Diagnostics and Targeted Interventions

### 7.1. Early Biomarkers

Early diagnosis of PD is increasingly focused on gut microbiota alterations that emerge during the long prodromal period. Research indicates that the microbial profile of patients with REM sleep behavior disorder (RBD), considered a prodromal stage of PD, shifts significantly toward that observed in early PD, characterized by a depletion of butyrate-producing bacteria and an overabundance of genera such as *Collinsella*, *Desulfovibrio*, and *Oscillospiraceae* UCG-005 [69]. These fecal microbiota communities differ significantly from those of controls not only in patients with RBD and early PD but also in their first-degree relatives (RBD-FDR), suggesting that these changes may represent early hallmarks of synucleinopathy [70].

The question of whether screening for constipation is a viable diagnostic tool is supported by underlying biological mechanisms. Bacteria belonging to the *Desulfovibrio* genus produce both LPS and H_2_S. While LPS has been associated with intestinal hyperpermeability and the accumulation of pathological α-synuclein, elevated H_2_S concentrations may inhibit gastrointestinal motility, leading to constipation [70]. Given that constipation is highly prevalent among PD patients and often precedes motor symptoms, changes in DSV content could serve as an effective indicator for predicting PD progression [17].

Furthermore, specific microbial signatures correlate with the severity of gastrointestinal symptoms. For instance, a higher relative abundance of *Desulfovibrionaceae*, *Faecalitalea*, and *Mogibacterium* is associated with defecation difficulties and severe constipation, whereas the abundance of genera such as *Lachnoclostridium* tends to be lower in these patients [71]. Although it remains unclear whether DSV species are a cause or a consequence of gastrointestinal dysfunction, they likely may contribute to the evolution of PD once their quantity exceeds a certain threshold [32]. Consequently, integrating the clinical assessment of constipation with microbiota-based biomarkers offers a promising avenue for early PD screening.

The clinical utility of these microbial signatures relies on understanding inter-community functional correlations. The expansion of *Desulfovibrio* represents a broader shift in microbial composition and abundance, typically accompanied by the depletion of beneficial, butyrate-producing communities [69,70]. Functionally, excessive H_2_S production and metabolic competition shift the gut microenvironment from anti-inflammatory saccharolytic fermentation to pro-inflammatory, sulfur-reducing pathways [18,68,72]. Consequently, both broad ecological interventions—such as dietary modulation and FMT—and targeted therapies like bacteriophages must aim beyond merely eradicating *Desulfovibrio* to reversing these dysfunctional ecosystem dynamics and restoring a neuroprotective metabolic balance [73,74].

### 7.2. Dietary Modulation

Dietary and supplemental interventions are emerging as promising strategies for modulating the gut microbiota and mitigating the pathogenesis of PD, particularly with respect to the genus *Desulfovibrio*. Recent research has identified a novel bacterial transaldolase pathway in which select microbes, including *Desulfovibrio* and *Eubacterium*, ferment dietary sulfoquinovose (SQ), a sulfur-containing metabolite abundant in leafy greens, into H_2_S via the intermediate 2,3-dihydroxypropane-1-sulfonate (DHPS) [75]. Although these pathways exist in healthy individuals, increased flux through this cryptic sulfur metabolic pathway may lead to excess H_2_S, contributing to mitochondrial dysfunction, oxidative stress, and gut dysbiosis associated with neurodegenerative diseases [76].

To counteract these pathological shifts, specific dietary supplements have shown promise in restructuring the gut microbial environment and inhibiting *Desulfovibrio* overgrowth. For instance, polysaccharides extracted from *Ulva lactuca* (UPE) have been found to reshape the gut microbiota in PD models by significantly reducing the abundance of *Desulfovibrio* while simultaneously mitigating neuroinflammation and dopaminergic neuron loss through the Nrf2/HO-1 and TLR4/NF-κB signaling pathways [77]. Similarly, sodium butyrate (NaB) supplementation has been shown to ameliorate motor deficits and α-synuclein pathology by regulating gut microbiota dysbiosis and restoring short-chain fatty acid (SCFA) metabolism [72]. The neuroprotective effects of NaB have also been attributed to its ability to inhibit the TLR4/MyD88/NF-kB signaling pathway, thereby reducing inflammation along the GBA [78]. Together, these findings suggest that targeting sulfur-metabolizing bacteria like *Desulfovibrio* through dietary polysaccharides and SCFAs may represent a potent therapeutic strategy for PD.

### 7.3. Precision Medicine

The therapeutic potential of fecal microbiota transplantation (FMT) and targeted interventions like bacteriophages in PD remains a subject of intense investigation, showing a clear divide between promising preclinical results and complex clinical realities. Across multiple mouse models, FMT has demonstrated the ability to correct gut dysbiosis, specifically reducing the overgrowth of *Desulfovibrio* and *Akkermansia*, thereby ameliorating gastrointestinal dysfunction and motor deficits [74]. These animal studies suggest that FMT from healthy donors can restore BBB integrity, suppress neuroinflammation via the TLR4/MyD88/NF-κB signaling pathway, and enhance mitochondrial antioxidant capacity through the AMPK/SOD2 pathway [74,79]. Conversely, transplanting microbiota from PD patients into mice has been shown to aggravate neurodegeneration and motor impairments, reinforcing the idea that an altered gut microbiome is a significant risk factor for the disease [79].

Despite these successes in the lab, clinical outcomes in humans have been less conclusive. A randomized clinical trial found that although FMT was generally safe, it did not provide clinically meaningful improvements in PD symptoms compared with placebo, with dysbiosis often resolving more frequently in the placebo group after bowel cleansing [80]. This discrepancy highlights the need for more rigorous, large-scale experiments to validate the reliability of FMT in a clinical setting [81]. Furthermore, the complexity of gut dysbiosis suggests that broad transplants may not be the only solution, a more precise approach could involve *Desulfovibrio*-specific bacteriophages. These phages could selectively target and eliminate pathogenic strains such as *Desulfovibrio vulgaris* while preserving beneficial commensal bacteria necessary for gut health, offering a surgical alternative to the total microbial overhaul of FMT [73].

## 8. Critical Discussion and Future Directions

### 8.1. Correlation vs. Causation

Recent research suggests that *Desulfovibrio* may play a significant role in the development of Parkinson’s disease. The presence of DSV in the gut microbiome of patients with PD was higher than in healthy individuals in the control group. A correlation has also been demonstrated between DSV levels and the severity and progression of PD symptoms [32].

Mechanisms that may contribute to the development of PD in patients with an overabundance of DSV in the microbiome include the production of hydrogen sulfide and polysaccharides, as well as the synthesis of magnetite by several strains, which likely induces the oligomerization and aggregation of α-syn protein [32].

These findings suggest that a possible mechanism underlying PD involves disturbances in the gut microbiome leading to DSV overgrowth and, consequently, to the development of PD. At the same time, a comparison of changes in microbiota composition in patients without PD but suffering from constipation, relative to healthy individuals, indicates significantly higher levels of *Desulfovibrio* spp. in patients with constipation. In these patients, elevated levels of *Cristensenellaceae* and *Firmicutes* were also observed. In vivo studies have also demonstrated that members of the genus *Desulfovibrio* slow intestinal transit [34].

Therefore, the question of whether DSV, once a certain threshold is crossed, contributes to PD development, or whether its progression is a result of motor disturbances (particularly constipation) characteristic of early-stage PD, remains unresolved and points to the need for further research [32].

### 8.2. Genetic Susceptibility

Studies have repeatedly confirmed that the composition of the microbiota differs in patients with PD compared with healthy individuals [68].

Research in animal models has confirmed this association. Transferring microbiota derived from individuals with PD to genetically susceptible mice (α-synuclein overexpressing; ASO) promoted enhanced α-syn-dependent motor deficits. Meanwhile, no significant effect on motor function was observed in wild-type (WT) recipient animals colonized with microbiota from any donor group, including PD donors. It was also shown that the abundance of many bacterial genera changes in ASO mice, but not in WT mice, receiving microorganisms from the same donor. The results of these studies in a preclinical mouse model support the hypothesis that PD microbiota contributes to disease symptoms in genetically susceptible hosts. Extrapolating these preclinical findings to humans may reinforce the concept that gene-microbiome interactions represent a previously unrecognized etiology of PD [35].

Meta-analyses combining microbiological assessment with genetic analysis and machine learning may offer new insights into the causes, early diagnosis, and treatment strategies for PD. A meta-analysis of the gut microbiome in PD, encompassing 2269 samples analyzed using the 16S rRNA gene and 236 samples using shotgun metagenomics, indicates increased relative abundance of potential pro-inflammatory bacteria, genes, and pathways in PD, while anti-inflammatory bacteria, genes, and pathways were significantly reduced. The study identified five bacterial genera with reduced abundance in PD patients (*Roseburia*, *Faecalibacterium*, *Blautia*, *Lachnospira*, and *Prevotella*) and five bacterial genera with increased abundance (*Streptococcus*, *Bifidobacterium*, *Lactobacillus*, *Akkermansia*, and *Desulfovibrio*). These changes may result in reduced levels of SCFAs, which may have anti-inflammatory effects, and increased levels of lipopolysaccharides, H_2_S, and glutamate, which may have pro-inflammatory effects. The authors point out that random forest models (RF) can predict PD with high accuracy based on 11 types (>80%) or 6 genes (>90%) associated with inflammation [68].

### 8.3. Proposed Research Roadmap

Available studies assessing the prevalence of DSV in patients with PD show a clear correlation between DSV abundance and PD prevalence and severity. These studies focus on evaluating only a few species of DSV. The authors point to the possibility that patients with low levels of the DSV species included in the study may have high levels of other, unassessed DSV species [32].

Huynh et al. demonstrated that bacteria isolated from stool samples of PD patients were more capable of inducing alpha-syn aggregation than DSV isolated from samples taken from healthy individuals [16].

Mohammadi et al. reached similar conclusions, demonstrating that *D. desulfuricans* DSM 6949 and *D.* spp. MUU 26 (a PD patient-isolate) induced higher levels of α-syn aggregation than an environmental strain [52].

It is estimated that in 20% of PD patients, the DSV species is unknown; therefore, these bacteria should be isolated and characterized to enable the development of primers suitable for qPCR [32].

The results indicate that DSV strains isolated from PD patients and healthy individuals appear to have different characteristics. At present, there are no clear guidelines describing how to distinguish DSV strains characteristic of PD. Therefore, the need for comparative genomics studies is highlighted in order to identify genetic differences and pathogenic genes in patients with PD and DSV [16].

## 9. Conclusions

The conceptualization of Parkinson’s disease pathogenesis is gradually evolving, with accumulating evidence suggesting that the disease process may, at least in a subset of patients, originate within the enteric nervous system. This review highlights the potential role of *Desulfovibrio* species as pathobionts that may participate in the proposed gut-first cascade. Through their sulfate-reducing metabolism, these bacteria may contribute to elevated H_2_S production. This metabolic byproduct could, in turn, compromise intestinal mucosal barrier integrity, potentially interfere with mitochondrial cytochrome c oxidase, and promote localized oxidative stress, thereby creating an environment conducive to α-syn misfolding and aggregation.

The possible retrograde propagation of these conformationally altered proteins along the vagus nerve, according to a hypothesis supported by selected epidemiological vagotomy studies, suggests a potential link between intestinal dysbiosis and central neurodegeneration. However, establishing *Desulfovibrio* as a direct driver of the disease process needs further investigation, given that current scientific evidence remains largely correlational.

**Figure 1:**
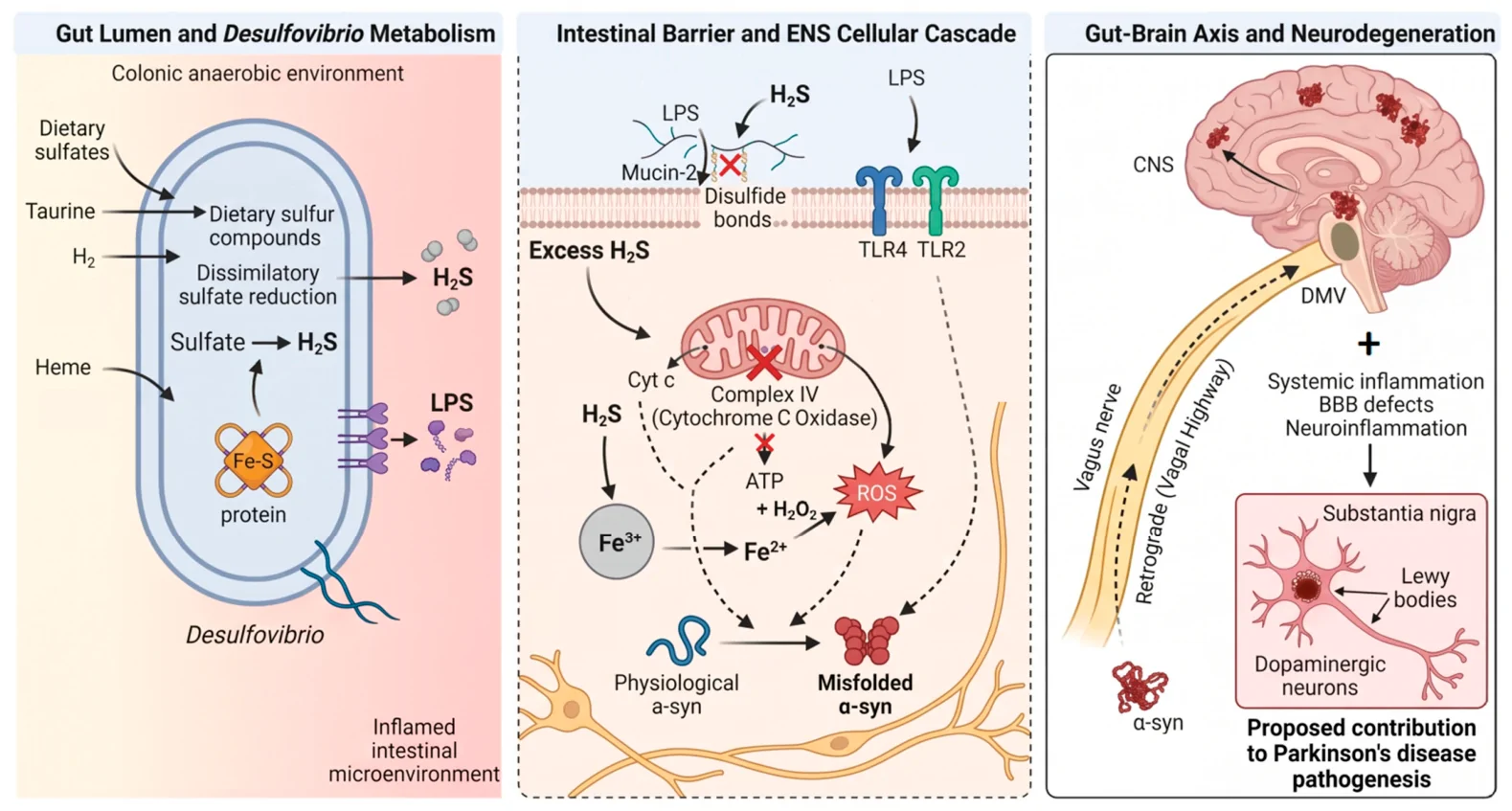
Proposed gut-to-brain cascade linking *Desulfovibrio* to Parkinson’s disease pathology.

Verification of these proposed mechanisms, together with a better understanding of host genetic susceptibility, requires rigorous investigation in advanced experimental models. Nonetheless, the available evidence supports further exploration of microbiome profiling in the context of prodromal PD diagnosis. Ultimately, targeted modulation of the intestinal environment whether through dietary interventions, enzymatic inhibitors, or specific bacteriophages represents an intriguing, albeit still preliminary, field of research for potentially delaying early-stage neurological decline. To synthesize these concepts, a graphical summary of the proposed gut-to-brain cascade linking *Desulfovibrio* to Parkinson’s disease pathology is presented in Figure 1.

## Figures and Tables

**Table 1:** Mechanistic Comparison of Microbiota-Driven Alpha-Synuclein Aggregation Models.

Feature	*Desulfovibrio* spp.	Curli-Producing *E. coli*	Other Pro-Inflammatory Bacteria
Primary Driver	Chemically and metabolically mediated mechanisms.	Structurally driven mechanism (structural templating and cross-seeding).	Inflammation-driven (insufficient to induce aggregation alone).
Key Bacterial Factors	Diffusible metabolites (predominantly H_2_S) and LPS.	Curli fibers (bacterial amyloids composed of CsgA and CsgB subunits).	General pro-inflammatory components.
Cellular Consequence	Mitochondrial dysfunction, elevated ROS (oxidative stress), altered iron metabolism, and impaired intracellular clearance.	Direct cross-seeding, accelerating α-syn fibrillization and deposition.	Chronic low-grade inflammation, immune activation, and increased intestinal permeability.
Strain Specificity	High; PD-derived isolates (e.g., *D. desulfuricans*, MUU 26) induce higher aggregation burden and larger aggregates than strains from healthy individuals.	High; strictly dependent on curli expression (curli-deficient strains do not induce aggregation).	Lack specific aggregation-promoting properties.
Main Evidence	*C. elegans* models show increased aggregates and mortality; metabolites can cross biological barriers to exert systemic effects.	In vivo experimental data show CsgA directly interacts with host amyloids to accelerate fibril formation.	Studies on *Proteus mirabilis* and *Klebsiella pneumoniae* show they promote inflammation but do not consistently trigger aggregation.

## Data Availability

No new data were created or analyzed in this study. Data sharing is not applicable to this article.
